# Phase I Trial of Lithium and Tretinoin for Treatment of Relapsed and Refractory Non-promyelocytic Acute Myeloid Leukemia

**DOI:** 10.3389/fonc.2020.00327

**Published:** 2020-03-10

**Authors:** Masumi Ueda, Tammy Stefan, Lindsay Stetson, James J. Ignatz-Hoover, Benjamin Tomlinson, Richard J. Creger, Brenda Cooper, Hillard M. Lazarus, Marcos de Lima, David N. Wald, Paolo F. Caimi

**Affiliations:** ^1^Clinical Research Division, Fred Hutchinson Cancer Research Center, Seattle, WA, United States; ^2^Department of Medicine, Division of Medical Oncology, University of Washington, Seattle, WA, United States; ^3^Department of Pathology, Case Western Reserve University, Cleveland, OH, United States; ^4^Stem Cell Transplant and Hematologic Malignancies Program, University Hospitals Seidman Cancer Center, Cleveland, OH, United States; ^5^Case Comprehensive Cancer Center, Case Western Reserve University, Cleveland, OH, United States

**Keywords:** AML, differentiation, stem cells, lithium, tretinoin

## Abstract

Glycogen synthase kinase-3 (GSK3) inhibitors induce differentiation and growth inhibition of acute myeloid leukemia (AML) cells. Our pre-clinical studies showed GSK3 inhibition leads to sensitization of AML cells to tretinoin-mediated differentiation. We conducted a phase I trial of lithium, a GSK3 inhibitor, plus tretinoin for relapsed, refractory non-promyelocytic AML. Nine patients with median (range) age 65 (42–82) years were enrolled. All subjects had relapsed leukemia after prior therapy, with a median (range) of 3 (1–3) prior therapies. Oral lithium carbonate 300 mg was given 2–3 times daily and adjusted to meet target serum concentration (0.6 to 1.0 mmol/L); tretinoin 22.5 or 45 mg/m^2^/day (two equally divided doses) was administered orally on days 1–7 and 15–21 of a 28-day cycle. Four patients attained disease stability with no increase in circulating blasts for ≥4 weeks. Median (range) survival was 106 days (60–502). Target serum lithium concentration was achieved in all patients and correlated with GSK3 inhibition in leukemic cells. Immunophenotypic changes associated with myeloid differentiation were observed in five patients. The combination treatment led to a reduction in the CD34+ CD38– AML stem cell population both *in vivo* and *in vitro*. The combination of lithium and tretinoin is well-tolerated, induces differentiation of leukemic cells, and may target AML stem cells, but has limited clinical activity in the absence of other antileukemic agents. The results of this clinical trial suggest GSK3 inhibition can result in AML cell differentiation and may be a novel therapeutic strategy in this disease, particularly in combination with other antileukemic agents. Lithium is a weak GSK3 inhibitor and future strategies in AML treatment will probably require more potent agents targeting this pathway or combinations with other antileukemic agents. This trial is registered at ClinicalTrials.gov NCT01820624.

## Introduction

Many acute myeloid leukemia (AML) patients are not eligible to receive initial or salvage standard cytotoxic chemotherapy due to advanced age, comorbidities or cumulative toxicity from prior therapy. Safe and effective treatments for this patient population are greatly needed. AML is defined by maturation arrest as well as rapid proliferation of leukemic cells. Currently available cytotoxic therapies primarily target the latter but not the maturation arrest. Inducing differentiation of leukemia cells is a potentially useful therapeutic approach that can force malignant cells to exit the cell cycle and decrease cell proliferation without overt toxicity. Such differentiation therapy has proven successful in the treatment of acute promyelocytic leukemia (1) but not in other AML subtypes. Prior studies have shown that glycogen synthase kinase-3 (GSK3) inhibition induces differentiation and proliferation arrest in non-promyelocytic AML cells *in vitro* and in pre-clinical animal models (2–7).

Although the mechanism by which GSK3 inhibition leads to AML cell differentiation is largely unknown, we previously reported that GSK3 inhibition can potentiate the differentiation effects of tretinoin (2, 8, 9). GSK3 is a constitutively active kinase that directly phosphorylates and impairs the activity and expression of tretinoin's receptor, the retinoic acid receptor (RAR) (2). Inhibition of GSK3 prevents this repression of RAR activity and leads to higher levels of tretinoin-mediated AML differentiation. Although various GSK3 inhibitors are available for *in vitro* testing, at present, lithium is the only clinically available drug that inhibits GSK3 (10–12). Lithium causes reversible enzyme inhibition of GSK3 via Serine-9 phosphorylation (12). We conducted a phase I trial of the combination of lithium and tretinoin in adult non-promyelocytic AML patients who were not candidates for standard induction chemotherapy or had relapsed or refractory disease.

## Patients and Methods

This investigation was a single-center study enrolling patients at University Hospitals Cleveland Medical Center in Cleveland, Ohio between May 2013 and August 2016. This study was approved by the Institutional Review Board for Human Investigation, and all patients gave written informed consent prior to enrollment. The study eligibility included patients age 18 years or older who had histologically or cytogenetically confirmed non-promyelocytic AML and whom based on judgement of the treating physician were unfit to receive standard intensive induction chemotherapy or who had relapsed or refractory disease. Eastern Cooperative Group (ECOG) performance status 0–2 was required. Prior treatment for pre-existing hematologic conditions was allowed, including hematopoietic cell transplantation. Concurrent use of hydroxyurea to control circulating blast counts was allowed during the study period. A minimum of 4 weeks was required from the administration of other prior conventional or investigational agents and patients with unresolved grade >1 treatment-related non-hematologic toxicities were excluded. All toxicities were graded according to the National Cancer Institute Common Toxicity Criteria (version 4.0), i.e., NCI CTCAE v 4.0. Patients were required to have adequate hepatic and renal function [total serum bilirubin ≤1.5 times institutional upper limit of normal (ULN), AST ≤2.5 times ULN, ALT ≤2.5 times ULN, and serum creatinine ≤1.5 times ULN]. Hematologic parameters were not a basis for exclusion. Exclusion criteria included central nervous system involvement by AML. Cerebrospinal fluid (CSF) analysis was not required and was only performed based on clinical suspicion by the treating physician.

### Treatment Protocol

**Figure 1:**
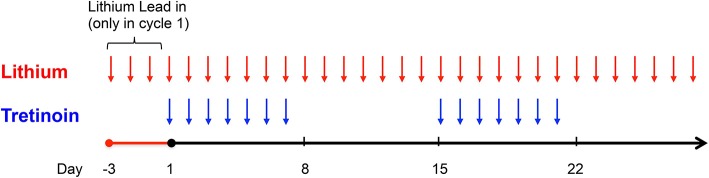
Study Schema. Lithium was administered for a 3-day “lead-in” period prior to administration of tretinoin in cycle 1 and thereafter given continuously. Tretinoin was dosed on days 1–7 and 15–21 of a 28-day cycle.

Lithium carbonate (lithium) 300 mg was administered orally three times a day (or two times daily for patients age ≥65 years) starting on day - 3 prior to cycle 1 and on day 1 of all subsequent cycles consisting of 28 days (Figure 1). The “lead-in” period prior to cycle 1 was designed to allow for correlative studies to investigate the effects of lithium on leukemic cell GSK3 activity and to ensure achievement of adequate serum lithium concentrations prior to tretinoin exposure. The target serum lithium concentration was the laboratory-defined therapeutic range of 0.6–1.0 mmol/L based on toxicity in humans for treatment of mood disorders. Dose adjustments were made based on the serum lithium concentration (Supplementary Figure 1). Dosing of tretinoin followed a “3+3” study design with three patients sequentially enrolled at each dose level (13). Tretinoin was administered orally at a dose of 22.5 mg/m^2^/day (dose level 1) or 45 mg/m^2^/day (dose level 2) in two divided doses, from days 1 to 7 and from days 15 to 21 of a 28-day cycle. Further tretinoin dose escalation to 60 mg/m^2^/day (dose level 3) and 90 mg/m^2^/day (dose level 4) was outlined in the protocol if significant clinical activity of the combination was observed in the first two cohorts.

### Toxicity Grading and Definition of Maximum Tolerated Dose (MTD)

Toxicity was graded for severity according to the NCI CTCAE v 4.0. The definition of dose limiting toxicities (DLT) included grade 3 or 4 non-hematologic toxicities attributed to lithium or tretinoin, with the exception of toxicities occurring in the context of tretinoin-induced differentiation syndrome (14) and toxicities considered to be directly associated with an elevated serum lithium concentration (15). Infectious complications and hospitalizations were not considered DLT, consistent with previously published studies in AML (16). The maximum tolerated dose (MTD) was determined based on toxicities observed during the first cycle of treatment. MTD was defined as the highest dose level at which ≤33% of patients experience DLT. Serious adverse events (SAEs) were defined as any life-threatening grade 3 or 4 non-hematologic toxicity or any toxicity requiring inpatient hospitalization or resulting in permanent disability.

### Response Criteria

Disease response was not a primary endpoint in this study, but evaluated based on previously published response criteria (17, 18). Subjects receiving at least 2 cycles of study protocol were evaluable for response by these criteria. Lack of response was not an indication to stop treatment in the absence of observed toxicity, and discontinuation of treatment was based on discretion of the treating physician. Survival was measured from the start of treatment (first dose of lithium) to death from any cause. Bone marrow biopsies were interpreted by hematopathologists at University Hospitals Cleveland Medical Center.

### Statistical Analysis

Survival was calculated using the Kaplan-Meier estimate. Patients not known to have died at last follow-up were censored on the last date they were known to be alive. For correlative studies, comparison of means between two groups or time points was performed using two-sample *t*-test assuming equal variances. Univariate repeated measures analysis of variance (ANOVA) was used to compare group means of continuous variables over the time course of the study. One-way ANOVA was used to compare means across treatment groups for the *in vitro* correlative analysis.

### Correlative Studies

Blood samples were collected at baseline, days 1, 8, and 30 and at the end of the study period. Specimens were processed only if ≥5% leukemic cells were present in the blood as assessed using morphologic criteria. Using this criterion, seven patients had blood samples evaluated for laboratory correlative studies. Bone marrow was collected at baseline and end of study. Immunophenotyping was performed by multi-parameter flow cytometry using fluorochrome-labeled antibodies to CD11b, CD14, and CD15 as markers for monocytic and/or granulocytic differentiation (19–22). The stained samples were run on a BD Biosciences LSRII (San Jose, CA, USA) flow cytometer. Cells were concurrently stained for CD34, CD117, and CD38 for identification of the leukemic blast and stem cell populations (CD34+ CD38–). After fixation and permeabilization using a formaldehyde-based solution, intracellular staining was performed for phosphorylated Serine-9 GSK3β (pGSK3β), the inactive or inhibited form of GSK3. Phosphorylated Serine-9 GSK3β antibody was obtained from Cell Signaling Technologies (Beverly, MA, USA).

In order to study the separate effects of GSK3 inhibition or tretinoin on AML stem cells, we used previously obtained cryopreserved blood samples from the Eastern Cooperative Oncology Group (ECOG) tumor repository of newly diagnosed and untreated AML patients (independent of this trial) to conduct *in vitro* studies. All patients previously gave written informed consent to the respective institutions at time of E1900 or E3999 study enrollment for participation of therapy and blood sample collection. Cryopreserved cells were thawed and cultured in RPMI 1640 media (Hyclone) containing 10% fetal bovine serum (Sigma-Aldrich, St. Louis, MO, USA) and 1% final concentration penicillin and streptomycin. Amphotericin B (0.5 μg/ml) and ciprofloxacin (10 μg/ml) were added to help eliminate fungal and mycoplasma contamination in the cell culture. Cells were treated with SB415285 (10 μM) obtained from Tocris (Bristol, UK), a laboratory-grade GSK3 inhibitor which competitively binds to the ATP binding site of GSK3 (23), and tretinoin (0.5 μM). SB415285 was used as a more potent GSK3 inhibitor than lithium to study the independent effects of GSK3 inhibition on the AML stem cell population. After 6 days, immunophenotyping for the CD34+ CD38– AML stem cell population was performed by flow cytometry.

## Results

### Patient Characteristics

Between May 2013 and August 2015, 12 patients (nine men and three women) were enrolled. Three patients enrolled in dose level 1 were considered not evaluable for DLT as they did not complete a full cycle of therapy. One subject discontinued study participation due to personal choice after 10 days, without observed toxicity. The second patient had rapidly progressive disease with high circulating blast counts and came off study at 12 days. The third patient had mast cell leukemia, a rare variant of AML, and was enrolled in the study after intolerance to initial therapy with imatinib. He had biopsy-proven gastrointestinal infiltration with mast cells, and had progressive disease characterized by gastrointestinal hemorrhage secondary to histamine release (plasma histamine level >6 times normal limit) after receiving only 9 days of study treatment. These patients were replaced with 3 additional patients. Median age of the remaining nine evaluable patients was 65 (range 42–82) years. Baseline patient characteristics are summarized in Table 1. All nine patients were previously treated for AML and had received a median of 3 (range 1–3) prior therapies. Two patients had relapsed disease after undergoing allogeneic hematopoietic cell transplantation. Two patients (Patient 5 and 9) received hydroxyurea for control of leukocytosis during the study period.

**Table 1:** Baseline patient characteristics.

**Patient**	**Age**	**Sex**	**Secondary AML**	**Cytogenetics/Molecular**	**# Prior therapy lines**	**Baseline marrow blasts (%) by morphology**	**Prior transplant**
1	82	M	No	46XY	1	32	No
2	42	M	No	t(17;22), trisomy 12	3	67	No
3^*^	65	F	No	46XX; *NPM1* mutation	2	19	Yes
4^*^	68	M	Yes	Monosomy karyotype	2	27	No
5	56	F	Yes	46XX; FLT3 ITD	3	NA	Yes
6^*^	72	M	No	46XY	1	50	No
7	65	M	No	Culture failure	3	3	No
8	72	M	No	46XY	2	39	No
9	73	F	No	Culture failure; *NPM1* mutation	2	64	No
10	58	M	No	Monosomy karyotype	2	23	Yes
11	72	M	No	46XY	3	18	No
12	49	M	No	46XY; *IDH2* mutation	3	57	No

* *Patients removed from analysis due to short duration on treatment; NA, not available*.

### Toxicity

Patients who received at least one dose of protocol therapy were evaluable for toxicity. One patient enrolled in dose level 1 presented with delirium and confusion during his third week of treatment. His confusion required hospitalization (CTCAE grade 3) and was attributed possibly to lithium toxicity, although the patient had underlying dementia and recent exposure to psychotropic medications including benzodiazepine, diphenhydramine, and steroids. The serum lithium concentration at admission was below the reference range for toxicity (0.28 mmol/L). The patient's mental status improved within 48 h after withholding all potential offending medications, including lithium. This neurologic event was considered a DLT. Dose level 1 was subsequently expanded to a total of six patients; no additional central nervous system toxicity or DLTs were observed. Three patients were enrolled at dose level 2 with no DLT observed. Adverse events are summarized in Table 2. With exception of the case of delirium, all grade 3–4 adverse events were attributed to progressive AML. Grade 3–4 hematologic toxicites were observed in all participating subjects, as expected in the setting of underlying AML and progressive disease. Two deaths occurred within the first 60 days of study protocol, both due to disease progression. No patients developed acute differentiation syndrome.

**Table 2:** Treatment emergent adverse events.

**Adverse events in subjects treated with lithium and tretinoin**				
**Toxicity**	**All grades (%)**		**Grade 3–4 (%)**	
**Hematologic**	***N***	**%**	***N***	**%**
Leukopenia	8	89	7	78
Neutropenia	7	78	5	56
Anemia	9	100	0	0
Thrombocytopenia	9	100	9	100
**Gastrointestinal**				
Anorexia	3	33	0	0
Nausea	1	11	0	0
Constipation	1	11	1	5
Diarrhea	1	11	0	0
**Neurologic**				
Confusion	1	11	1	11
Dizziness	1	11	0	0
Headache	2	22	0	0
**Respiratory**				
Cough	1	11	0	0
Dyspnea	1	11	0	0
**General**				
Fatigue	5	56	0	0
Myalgias	2	22	0	0
**Cutaneous/Dermatologic**				
Rash	1	11	0	0
Pruritus	1	11	0	0
Infectious				
Fever	6	67	1	5
Infection	2	22	2	22
**Laboratory**				
Transaminase elevation	2	22	0	0
Creatinine elevation	2	22	0	0

### Response and Outcomes

The median duration on treatment protocol for the nine patients was 55 (range 31–112) days. The median number of cycles of therapy was 2 (range 1–4). Response to treatment was assessed after 2 cycles or at the time of removal from study. No patient achieved hematologic improvement using standard AML response criteria. Disease progression occurred in all nine patients. Four patients had a period of disease stability defined by absence of increase in circulating blasts for ≥4 weeks. Of these four patients, none had sustained improvement in other blood count parameters or significant improvement in transfusion requirement. Median survival was 106 (range 60–502) days for the nine evaluable patients. Treatment with hydroxyurea (*n* = 2) did not have an effect in disease response or survival.

One subject went on to receive further therapy with 5-azacytidine and subsequent allogeneic transplant. Because of the absence of significant hematologic response, further escalation of tretinoin was not explored in this trial, with plans for subsequent trials combining GSK3 inhibition with additional antileukemic therapy for increased efficacy or use of more potent GSK3 inhibitors.

### Pharmacodynamics and Correlative Studies

Correlative laboratory studies were performed to investigate the following: (1) *in vivo* GSK3 inhibition with lithium; (2) induction of myeloid differentiation of leukemia blasts; and (3) the effect of GSK3 inhibition on the AML stem cell population.

#### Serum Lithium Concentrations and GSK3 Inhibition

**Figure 2:**
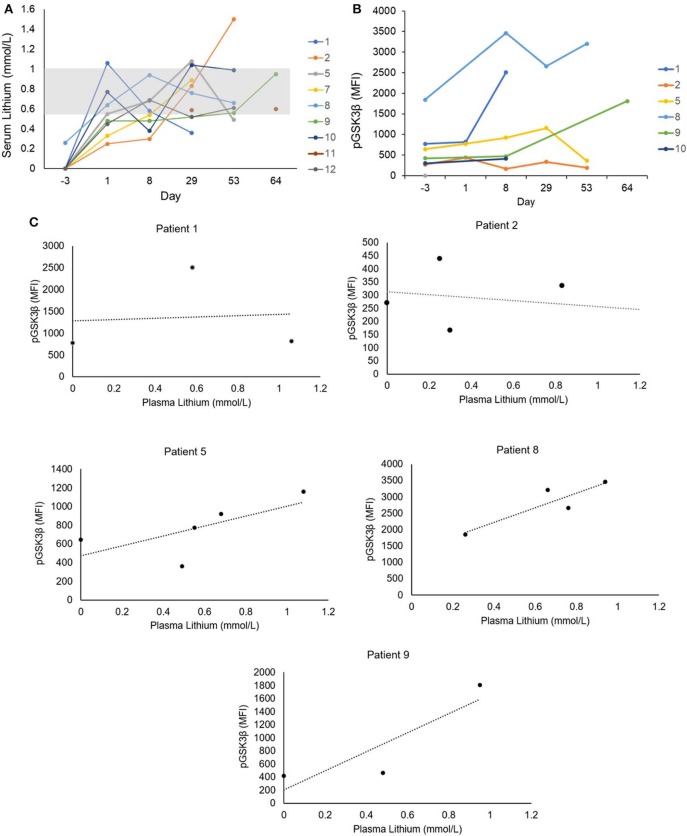
**(A)** Serum lithium levels. Serum lithium levels were measured throughout the study period. The clinical laboratory-defined serum lithium reference range according to toxicity is indicated by the gray box (0.6–1.0 mmol/L). **(B)** GSK3 inhibition in leukemia cells. Six subjects had blood samples with adequate numbers of leukemic cells available for analysis. Intracellular staining was performed for phosphorylated Serine-9 GSK3β (pGSK3β), the inactive form of GSK3. Stained cells were analyzed by flow cytometry and expression of pGSK3β in CD34+ and/or CD117+ cells was quantified by mean fluorescence intensity (MFI). **(C)** Correlation of serum lithium level and GSK3 inhibition. Five subjects had at least 3 time points available for analysis of pGSK3β. A positive correlation between serum lithium concentration and GSK3 inhibition measured by pGSK3β was observed in four subjects.

The laboratory-defined target serum lithium concentration (0.6–1.0 mmol/L) was achieved at least once in all nine patients (Figure 2A); however, levels were highly variable and frequent dose adjustments were necessary. It has previously been reported that lithium threshold concentrations of ~0.8 mmol/L are necessary *in vivo* to significantly inhibit GSK3 (24). The inhibition of GSK3, as measured by increase in pGSK3β, was achieved to varying degrees in the six patients with evaluable samples (Figure 2B). Baseline pGSK3β was variable, and the median increase in pGSK3β measured as change in mean fluorescence intensity (MFI) from baseline was 1,093 (range, 111–1,738). Furthermore, a positive correlation between serum lithium concentration and inhibition of GSK3β (as measured by increase in pGSK3β) was observed. Of the five patients with evaluable samples for pGSK3β at a minimum of 3 time points, the median Pearson correlation coefficient was 0.69 (range −0.3 to 1). Two patients (Patient 5 and 8) exhibited strong correlation and had at least 4 time points available for analysis (Figure 2C).

#### Evidence for Myeloid Differentiation

We next evaluated for differentiation by measuring the increase in the percentage of cells expressing surface antigens associated with myeloid differentiation (CD11b, CD14, and CD15). This was measured as a fraction of all peripheral blood mononuclear cells (PBMCs) as well as specifically within the CD34 and/or CD117-positive blast population. Analysis of both of these compartments allowed for assessment of the total population of cells, including leukemia blasts that may have lost CD34 and/or CD117 expression with differentiation.

**Figure 3:**
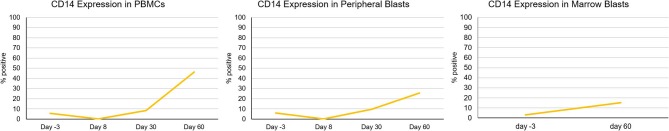
Increase in differentiation-associated immunophenotype in blood and marrow in a patient. Patient 8 showed a notable increase in percentage of cells expressing CD14, a surface marker associated with myeloid differentiation throughout the study period. Percent expression indicates fraction of cells expressing CD14 within CD45+ leukocytes (PBMCs), circulating blasts, or marrow blasts.

Seven subjects had samples evaluable for analysis (based on pre-defined criteria of ≥5% circulating blasts). Overall, changes in expression of the surface antigens associated with myeloid differentiation was variable throughout the study period (Table 3A). Nonetheless, five of the seven subjects (71%) showed ≥10% increase in cells expressing at least one of the surface antigens (CD11b, CD14, and/or CD15) with median increase of 22% (range 10–59%) within the PBMC compartment. Three subjects had ≥10% increase in at least one of the surface antigens associated with myeloid differentiation with median increase of 17% (range 17–38%) specifically in the blast cell compartment. Across the seven subjects with correlative samples, no statistically significant change in differentiation-associated surface antigen expression from baseline to end of study was observed using univariate repeated measures ANOVA. Although statistical significance was not achieved due to the low sample size, increase in the differentiation-associated phenotypic markers was observed in most patients at several different time points. Additionally, three patients had day 60 bone marrow samples available for correlative laboratory analysis; all three showed an increase in the expression of at least one differentiation-associated surface marker (CD11b, CD14, and/or CD15) within the CD34+ or CD117+ blast population (Table 3B). Patient 8 showed consistent increase in CD14 expressing cells in the blood and marrow throughout the study period (Figure 3).

**Table 3A:** Expression of differentiation markers in blood cells.

	**CD11b expression (%) in PBMCs**					**CD11b expression (%) in peripheral blasts**			
**Patient**	**Day −3**	**Day 8**	**Day 30**	**Day 60**	**Patient**	**Day −3**	**Day 8**	**Day 30**	**Day 60**
1	65.1	82.1	73.8	NA	1	6.0	2.6	3.2	NA
2	7.2	9.4	7.8	3.3	2	4.6	1.1	0.7	0.6
5	0.0	0.0	0.4	1.7	5	0.0	0.0	0.4	0.1
8	48.8	NA	39.9	18.7	8	0.2	NA	0.6	0.7
9	11.5	23.9	29.0	70.7	9	64.5	64.3	47.8	25.3
10	40.6	65.6	15.9	37.0	10	39.8	49.8	36.1	77.4
11	62.1	72.2	65.5	37.5	11	82.7	36.6	45.0	32.2
	**CD14 expression (%) in PBMCs**					**CD14 expression (%) in peripheral blasts**			
**Patient**	**Day −3**	**Day 8**	**Day 30**	**Day 60**	**Patient**	**Day −3**	**Day 8**	**Day 30**	**Day 60**
1	1.8	1.7	0.9	NA	1	0.2	0.5	0.0	NA
2	0.6	0.2	0.1	0.2	2	0.3	0.4	0.0	0.1
5	0.0	0.1	0.0	0.0	5	0.0	0.5	0.1	0.0
8	5.8	NA	8.4	46.2	8	6.1	NA	9.7	25.6
9	0.3	0.1	0.1	10.4	9	2.8	0.2	0.1	5.8
10	0.5	0.2	0.2	0.5	10	2.4	1.7	0.2	2.1
11	5.6	2.8	2.9	1.7	11	0.6	0.7	0.5	1.8
	**CD15 expression (%) in PBMCs**					**CD15 expression (%) in peripheral blasts**			
**Patient**	**Day −3**	**Day 8**	**Day 30**	**Day 60**	**patient**	**Day −3**	**Day 8**	**Day 30**	**Day 60**
1	64.1	77.6	69.9	NA	1	6.3	2.0	3.8	NA
2	14.5	8.6	1.7	6.0	2	4.8	2.7	1.8	14.4
5	0.1	1.0	0.5	1.6	5	0.7	0.5	3.1	1.0
8	57.3	NA	56.2	21.1	8	12.2	NA	6.6	7.6
9	4.4	19.1	35.9	17.9	9	42.8	16.2	11.9	12.6
10	33.2	55.3	2.4	12.1	10	13.7	13.5	2.9	15.0
11	46.8	55.7	49.7	28.1	11	4.3	14.5	21.5	9.4

*Percentage of cells expressing surface markers associated with myeloid differentiation (CD11b, CD14, CD15) in blood mononuclear cells and circulating blasts were examined throughout the study period. Percentages represent the mean value of duplicates. NA, sample not available*.

**Table 3B:** Expression of differentiation markers in marrow blasts.

	**CD11b**		**CD14**		**CD15**	
**Patient**	**Day −3**	**Day 60**	**Day −3**	**Day 60**	**Day −3**	**Day 60**
2	9.3	25.0	NA	NA	4.1	36.1
8	4.8	2.4	3.0	15.2	45.0	14.8
11	8.9	38.2	3.8	6.6	20.7	14.7

*Percentage of marrow blasts expressing surface markers associated with myeloid differentiation (CD11b, CD14, CD15). Percentages represent the mean value of duplicates. NA, sample not available*.

#### Impact on the AML Stem Cell Population

**Figure 4:**
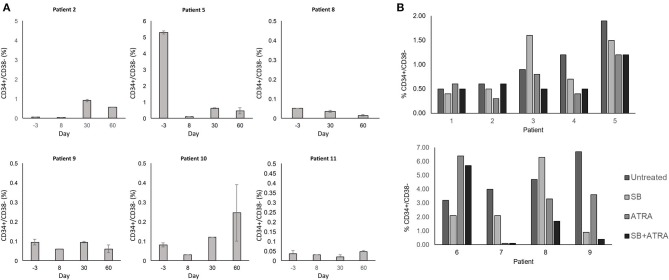
**(A)** Effect of treatment on the CD34+ CD38- stem cell population. The CD34+ CD38− population in the blood was monitored by flow cytometry throughout the study period. Percent expression indicates fraction of CD34+ CD38− cells within all PBMCs. Error bars indicate standard error of mean (SEM) of technical duplicates. **(B)** *In vitro* treatment of primary AML cells and impact on stem cell population. Cryopreserved blood samples from AML patients were thawed, and cells in culture media were treated with SB415285 (10 μM) and ATRA (0.5 μM). After 6 days, cells were stained for surface markers and analyzed by flow cytometry for analysis of the CD34+ CD38− population. Percent expression indicates fraction of CD34+ CD38− cells within all PBMCs. Technical replicates were not performed due to limited clinical samples.

Lastly, we investigated the potential for our therapeutic approach to target the AML stem cell population as these cells are poorly targeted by current AML therapies and play a key role in disease relapse (25, 26). We measured the CD34+ CD38– cells in the blood as a population enriched for AML stem cells. We did not observe a significant trend of decrease in AML stem cell population in our limited sample size (Figure 4A). However, in the 6 patients with evaluable samples, the median relative reduction from baseline (day −3) to the nadir proportion of CD34+ CD38– cells during the study period was 67% (range 37–98%). Two of the three patients with baseline and day 60 bone marrow correlative analyses (patients 8 and 11) presented a decrease in the CD34+ CD38– population, with relative reduction of 78 and 50% in the bone marrow CD34+ CD38– population, respectively. Interestingly, patient 8 who had the most notable increase in differentiation surface markers (Figure 3) also showed decrease of the CD34+ CD38 population in the blood and marrow.

To examine further whether the combination of tretinoin and lithium exhibited effects on AML stem cells, we performed *in vitro* studies to assess whether this effect was due to tretinoin-mediated GSK3 inhibition, or the combination. These studies were performed using cryopreserved blood samples from newly diagnosed, untreated AML patients that were obtained independently of this trial. After thawing, these cells were cultured and treated for 6 days with a laboratory-grade GSK3 inhibitor SB415285, tretinoin, or the combination. SB415285, a more potent GSK3 inhibitor than lithium, was chosen to study the specific effect of GSK3 inhibition on AML stem cells. The combined *in vitro* treatment with SB415285 and tretinoin resulted in a greater decrease in the CD34+ CD38– AML cell population compared to either agent alone in three of nine samples tested (Figure 4B). In three samples, treatment with tretinoin alone and in combination with SB415285 showed comparable reduction of the CD34+ CD38– population (Figure 4B). These results suggest that the combination of tretinoin with a GSK3 inhibitor is effective in targeting AML stem cells, although the sensitivity of the stem cells to either agent alone or their combination is variable from patient to patient, likely due to the heterogeneity of AML. Overall our results suggest that the combination of GSK3 inhibition and tretinoin can target the CD34+ CD38– AML stem cell population both *in vitro* and in human subjects.

## Discussion

We describe a novel approach using lithium, a GSK3 inhibitor, in combination with tretinoin for differentiation therapy in relapsed, refractory AML patients. In our cohort of older AML patients who had received a median of three lines of prior therapy, our regimen was well-tolerated and showed evidence of inducing myeloid differentiation. Furthermore, lithium treatment resulted in some degree of GSK3 inhibition in all studied patients at clinically achievable serum lithium concentrations. Finally, our approach appears to target AML stem cells.

One DLT was observed in our phase I study: a case of delirium in an elderly patient with underlying dementia. Although the serum lithium levels were low, the event was considered as possibly related to lithium, as central nervous system abnormalities can occur even with sub-therapeutic lithium serum concentrations, especially in elderly subjects. Hematologic adverse events were common, although these toxicities were most likely secondary to persistent and progressive AML. No other severe adverse events were attributable to the combination of lithium and tretinoin, with all other reported serious adverse events considered secondary to progressive disease. The two deaths that occurred within the first 60 days of treatment were secondary to disease progression. While the combination of tretinoin and lithium appeared safe, further dose escalation beyond 45 mg/m^2^ was not pursued as there was no significant clinical response. The rationale behind this decision was that a combination of differentiation agents and cytotoxic agents would be necessary to control rapidly proliferating leukemia cells while allowing for differentiation of remaining disease. Four patients (44%) exhibited disease stability while on the study regimen, defined by the absence of increase in the circulating blast count for ≥4 weeks. While all patients eventually had progressive disease, the disease stability observed in a proportion of the study population serve as a basis for further investigation of GSK3 inhibition as a therapeutic approach for AML. Furthermore, this therapeutic approach may be more effective in patients with minimal residual disease following conventional chemotherapy and warrants further study in this setting.

The variability in the baseline levels of GSK3 activity is likely a reflection of the heterogeneity of the disease. The clinical significance of the degree of change in GSK3 inhibition with therapy is difficult to interpret in light of highly variable serum lithium levels throughout the study period. Overall, the serum lithium levels achieved were at the lower end of concentrations that have been shown to inhibit GSK3 *in vivo* (24), likely leading to the observed heterogeneity in the degree of *in vivo* GSK3 inhibition. Nonetheless, it is notable that all patients analyzed showed a detectable increase in pGSK3β levels with therapy. Future studies in a larger number of patients will allow for correlative analysis between baseline pGSK3β or degree of change in pGSK3β and clinical response.

We observed phenotypic changes associated with myeloid differentiation in the setting of GSK3 inhibition that correlated with serum lithium levels. The degree of increase in the cell fractions expressing differentiation-associated surface antigens was variable among patients, most likely a reflection of the biologic heterogeneity of AML. Distinct leukemia biology and variable clinical conditions such as infection likely contributed to the observed differences. The response to GSK3 inhibition is also likely to be patient-specific. Most patients did not have a sustained trend of increased differentiation phenotype throughout the study period likely due to progressive disease. Additionally, leukemia cell immunophenotype within a single patient is likely to be heterogeneous, secondary to the presence of multiple clonal populations, and may also change over time with disease progression (27, 28). Despite the observed variability within and across patients, overall our correlative studies suggest the induction of myeloid differentiation with the combination of tretinoin and lithium.

Most notably, unlike most cytotoxic AML therapies which target rapidly dividing cells, our therapeutic strategy may be effective in targeting the AML stem cell population. All patients with evaluable samples for analysis showed a relative reduction in the CD34+ CD38– population in the blood. Moreover, *in vitro* correlative studies further demonstrated that this therapeutic regimen has high promise for targeting AML stem cells. While a more definitive evaluation of the effect on the stem cell population will require further work, our results support the future investigation of combining GSK3 inhibition and tretinoin in depleting the AML stem cell population.

Although this study demonstrated the ability to inhibit GSK3 in leukemic blasts and induce AML cell differentiation, it is possible that higher levels of GSK3 inhibition are necessary for greater clinical efficacy. As lithium concentrations cannot be increased significantly without incurring drug toxicity, future studies using more potent GSK3 inhibitors in AML are needed to further investigate this strategy. Several GSK3 inhibitors have been investigated in early-phase clinical trials for malignant (29–31) and non-malignant disorders (32, 33). For example, LY2090314 is a potent and competitive inhibitor of GSK3 (GSK3α IC_50_ = 1.5 nM; GSKβ IC_50_ = 0.9nM) with high specificity (34) that has been studied in phase I and II studies (29, 30). In addition, novel GSK3 inhibitors with more potent AML differentiation activity *in vitro* are under pre-clinical development (9). Future studies combining these agents with tretinoin are planned.

In conclusion, our phase I study showed that the combination of lithium and tretinoin is safe and well tolerated and induces leukemic cell differentiation in an older, heavily-pretreated cohort of AML patients with relapsed, refractory disease. Lithium, at clinically attainable serum concentrations, achieved modest GSK3 inhibition. The combination treatment results in *in vivo* and *in vitro* reduction of the AML stem cell population, an unmet need in the field of AML therapeutics. Differentiation therapy in patients with minimal residual disease or low leukemia burden following conventional chemotherapy warrants further investigation. More potent GSK3 inhibitors are likely necessary for greater clinical efficacy and combinations of GSK3 inhibition with cytotoxic therapies may achieve greater clinical activity.

## Data Availability Statement

All datasets generated for this study are included in the article/Supplementary Material.

## Ethics Statement

The studies involving human participants were reviewed and approved by University Hospitals Cleveland Medical Center Institutional Review Board for Human Investigation. The patients/participants provided their written informed consent to participate in this study.

## Author Contributions

MU wrote the manuscript. MU, TS, and DW performed correlative studies. LS, JI-H, BT, RC, BC, HL, ML, DW, and PC critically revised the manuscript. PC designed and conducted the clinical trial.

### Conflict of Interest

The authors declare that the research was conducted in the absence of any commercial or financial relationships that could be construed as a potential conflict of interest.
