# Supplementary figures and images for: Phase I Trial of Lithium and Tretinoin for Treatment of Relapsed and Refractory Non-promyelocytic Acute Myeloid Leukemia
Source: Front Oncol. 2020 Mar 10;10:327. doi: 10.3389/fonc.2020.00327 (PMC7076174; doi:10.3389/fonc.2020.00327)

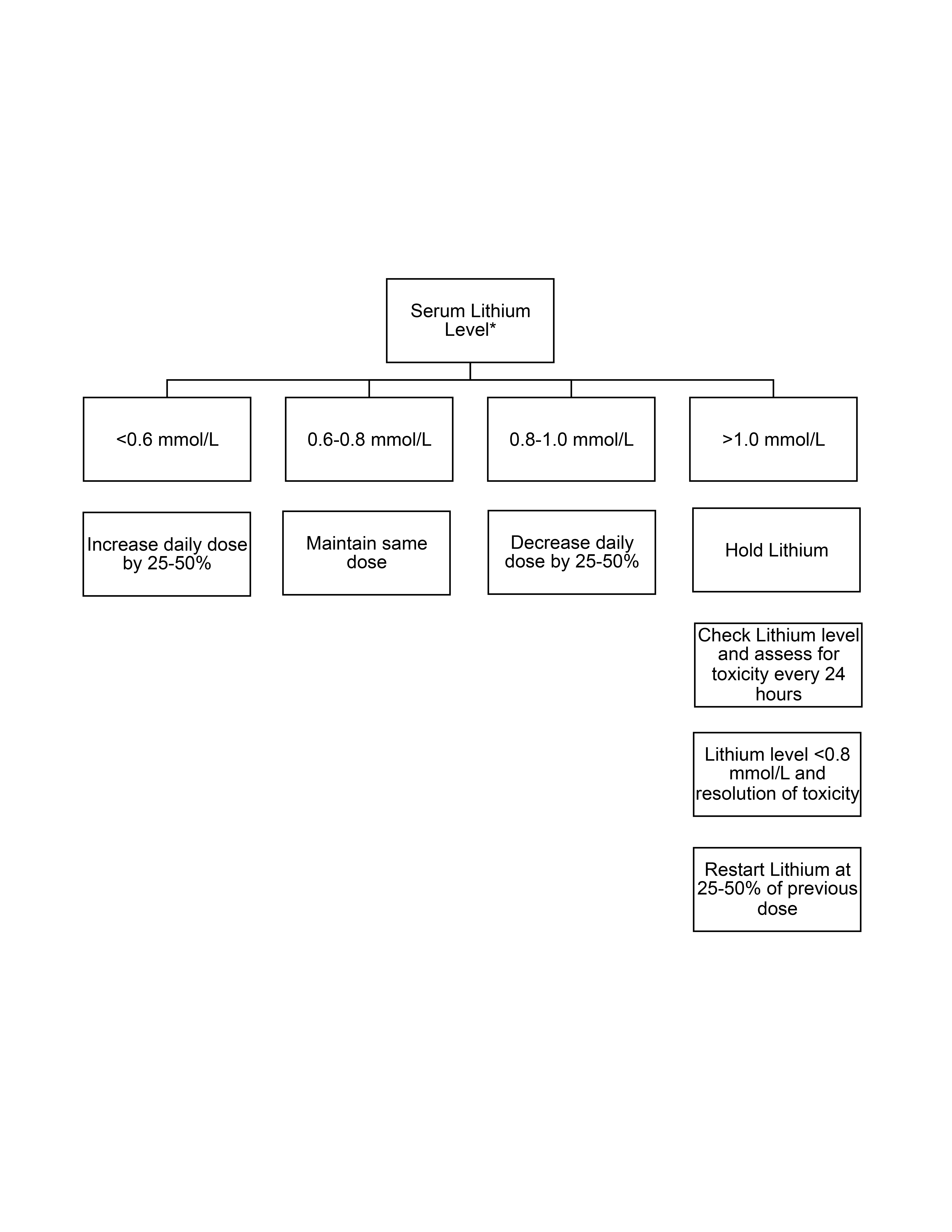

Supplement: Supplementary file 1 [file Image_1.TIF]
